# Ionic and Electronic Conductivities of Lithium Argyrodite Li_6_PS_5_Cl Electrolytes Prepared via Wet Milling and Post-Annealing

**DOI:** 10.3389/fchem.2021.778057

**Published:** 2021-12-16

**Authors:** Jae Min Lee, Young Seon Park, Ji-Woong Moon, Haejin Hwang

**Affiliations:** ^1^ Department of Materials Science and Engineering, Inha University, Incheon, South Korea; ^2^ Battery Materials Research Center, Research Institute of Industrial Science and Technology, Pohang, South Korea

**Keywords:** all-solid-state battery, lithium argyrodite, milling, conductivity, discharge capacity

## Abstract

Lithium argyrodite Li_6_PS_5_Cl powders are synthesized from Li_2_S, P_2_S_5_, and LiCl via wet milling and post-annealing at 500°C for 4 h. Organic solvents such as hexane, heptane, toluene, and xylene are used during the wet milling process. The phase evolution, powder morphology, and electrochemical properties of the wet-milled Li_6_PS_5_Cl powders and electrolytes are studied. Compared to dry milling, the processing time is significantly reduced via wet milling. The nature of the solvent does not affect the ionic conductivity significantly; however, the electronic conductivity changes noticeably. The study indicates that xylene and toluene can be used for the wet milling to synthesize Li_6_PS_5_Cl electrolyte powder with low electronic and comparable ionic conductivities. The all-solid-state cell with the xylene-processed Li_6_PS_5_Cl electrolyte exhibits the highest discharge capacity of 192.4 mAh·g^−1^ and a Coulombic efficiency of 81.3% for the first discharge cycle.

## Introduction

Inorganic solid electrolytes-based all-solid-state lithium-ion batteries (ASSLIBs), are expected to replace liquid electrolytes-based conventional lithium-ion batteries (LIBs) owing to their advantages, such as safety, higher energy density, and wider operation temperature range (Gao et al., 2018; Lee et al., 2019; Yu et al., 2021). Inorganic solid electrolytes are typically categorized into two groups: sulfide-based and oxide-based. Presently, the sulfides outperform oxides due to higher conductivity and facile manufacturing. A cold-pressed sulfide electrolyte has higher ionic conductivity than an oxide electrolyte sintered at high temperatures (Yu et al., 2018; Huang et al., 2021). Although sulfide-based electrolytes suffer from air instability, releasing toxic hydrogen sulfide (H_2_S) gas, their tolerance to moisture (hydrolysis resistance) is rapidly improving (Chen et al., 2015; Zhang et al., 2019; Zhao et al., 2020).

Sulfide-based solid electrolytes such as lithium argyrodite (Li_6_PS_5_Cl) are typically prepared by mechanical milling and subsequent annealing processes. The precursor materials (Li_2_S, P_2_S_5_, and LiCl) are dry-milled in a high-speed planetary ball mill, and the obtained powder mixture is annealed in a vacuum furnace or in an Ar-filled ampule to obtain a crystalline argyrodite phase. The lithium argyrodite electrolytes prepared via the aforementioned process exhibit a room temperature (25°C) ionic conductivity exceeding 10^–3^ S·cm^−1^ (Boulineau et al., 2012; Yu et al., 2016; Yu et al., 2017). However, the dry ball milling process is long and tedious (Boulineau et al., 2013; Yu et al., 2017; Strauss et al., 2020). Additionally, the precursors adhere to the milling jar wall or grinding media (normally, zirconia balls) because of their sticky nature, which can cause compositional and morphological inhomogeneity in the precursor powder mixture prior to the post-annealing process (Wang et al., 2020; Yu et al., 2021).

In wet ball milling, the precursors are mixed in non-dissolving organic solvents. Wet ball milling offer advantages over dry ball milling in terms of improved homogeneity, shortened duration, and scalability (Zhang et al., 2010). However, to the best of our knowledge, wet ball milling and post-annealing processes have not been employed extensively for the synthesis of sulfide-based electrolytes. This is probably due to insufficient understanding of the interaction between the precursors and solvents, and due to a cumbersome energy-intensive solvent evaporation step.

In this study, Li_6_PS_5_Cl solid electrolyte powders were synthesized via wet ball milling and post-annealing. Various non-polar organic solvents, such as hexane, heptane, toluene, and xylene, were employed as a liquid medium for high-energy planetary ball milling. The effect of the liquid medium on the phase evolution and powder morphology during the wet milling and post-annealing was investigated. In addition, the influence of solvents on the ionic and electronic conductivities of the Li_6_PS_5_Cl solid electrolyte pellets is also discussed.

## Materials and Methods

A stoichiometric amount of Li_2_S (99%, Sigma Aldrich), P_2_S_5_ (99%, Sigma Aldrich), and LiCl (98+%, Alfa Aesar) powders were mixed in a planetary ball mill (Pulverisette 6, Fritsch, Germany) using organic solvents such as hexane (96.0+%, deoxidized, Wako, Japan), heptane (99.0+%, super dehydrated, Wako, Japan), toluene (99.5+%, deoxidized, Wako, Japan), and xylene (super dehydrated, Wako). Typically, the milling was performed using 2 g of the powder mixture with zirconia balls (*d* = 5 mm) in a zirconia jar (80 ml) at a rotational speed of 500 rpm for a duration of 2 h. The slurry was vacuum dried at 150°C for 6 h followed by annealing at 550°C in a tube furnace for 4 h under an argon atmosphere. Argon flow rate was 50 sccm. For comparison, Li_6_PS_5_Cl powder was also synthesized via a dry ball milling process, details of which have been reported elsewhere (Park et al., 2021).

The phase analysis of the synthesized powder samples was performed using X-ray diffraction (XRD) (RU-200B, Rigaku Co. Ltd., Japan) with Ni-filtered Cu-Kα radiation. Surface chemical analysis was conducted via the X-ray photoelectron spectroscopy (XPS) (5400 ESCA, Perkin-Elmer, United States) using a Mg Kα (1,253.6 eV) excitation source. The carbon and hydrogen contents of the Li_6_PS_5_Cl powder were determined using an elemental analyzer (EA, Thermo EA1112, Thermo Fisher Scientific, USA). The microstructures were examined via field-emission scanning electron microscopy (FE-SEM) (JSM-6700F, JEOL, Japan).

The conductivities (ionic and electronic) were measured via electrochemical impedance spectroscopy (EIS) (SP-300, Biologic, France) over a frequency range of 0.01 Hz–1 MHz at 30, 40, 60, 80, 100, and 120°C. The Li_6_PS_5_Cl powder (55 mg) was placed in a zirconia mold (10 mm) equipped with two stainless-steel rods (*d* = 10 mm) and cold-pressed at 300 MPa to prepare a disk-shaped pellet sample (diameter = 10 mm, thickness ∼0.5 mm). The relative densities of the pellet samples were estimated to be approximately 92%, calculated from the bulk density of the electrolyte pellet sample and the theoretical density of Li_6_PS_5_Cl (1.64 g·cm^−3^) (Zhou et al., 2019).

The alternating current (AC) impedance spectra were obtained from the electrolyte pellet samples with two stainless-steel (SS) rods acting as current collectors under open-circuit conditions with an excitation potential of 20 mV. The total conductivity, σ_ionic+electronic_, was calculated using the equation σ_ionic_ = t/RA, where R is the total resistance, *t* is the thickness, and A is the area of the pellet sample, respectively. The ionic conductivities were determined by subtracting the electronic conductivities by DC polarization technique from the total conductivities.

The electronic conductivity was measured via direct current (DC) polarization technique (Boulineau et al., 2012; Zhang et al., 2020; Vadhva et al., 2021). The Ni/Li_6_PS_5_Cl/Ni symmetrical cells were prepared in a similar way as previously mentioned. Three voltages (0.25, 0.5, and 0.75 V) were applied to the Ni/Li_6_PS_5_Cl/Ni symmetrical cells for 2 h, and the resulting current signal was recorded. The resistances were determined from the V-I curves, and the electronic conductivity, σ_electronic_, was calculated from the thickness and area of the Li_6_PS_5_Cl electrolyte pellet sample.

All-solid-state cells (ASSCs) of LiNi_0.8_Co_0.1_Mn_0.1_O_2_ (NCM)/Li_6_PS_5_Cl/super P|Li_6_PS_5_Cl|Li-In were assembled by cold pressing at 300 MPa using a zirconia mold (*d* = 10 mm) equipped with stainless-steel rods. The ratio of NCM:Li_6_PS_5_Cl:super P was 70:29:1 by weight. The cathode loading of NCM was 26.8 mg·cm^−2^. Wet-milled and dry-milled Li_6_PS_5_Cl powders were used as the solid electrolyte and the composite cathode, respectively to exclude the solvent effect on reactions at the cathode/solid electrolyte interface. The charge–discharge behavior of the ASSCs was studied at 80°C using a battery test system (SP-300, Biologic, France) with a cutoff voltage of 1.9–3.6 V (vs Li–In). The current density was set to 0.535 mA cm^−2^. Charging and discharging were carried out in constant current (CC)–constant voltage and CC modes, respectively.

## Results and Discussion

**FIGURE 1:**
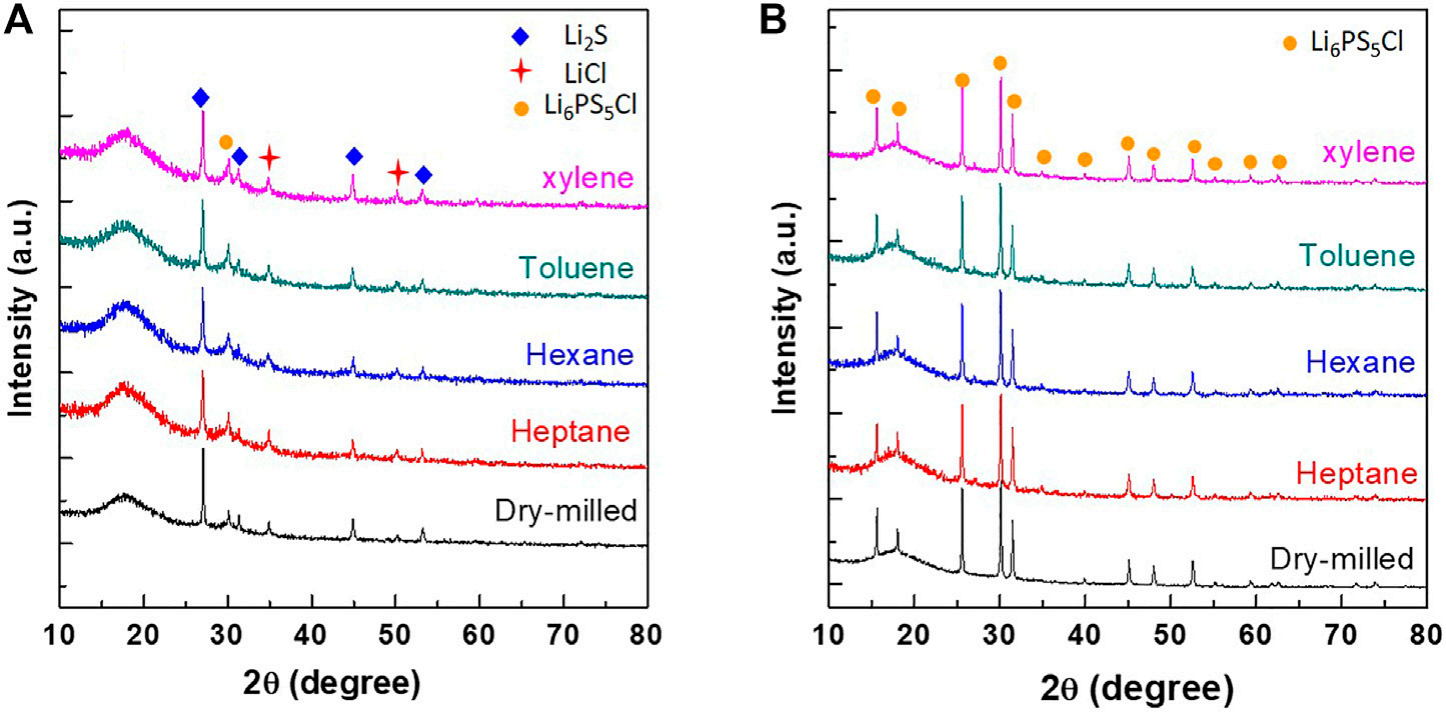
XRD patterns of dry- and wet-milled (hexane, heptane, toluene, and xylene) powder samples **(A)** before and **(B)** after post-annealing at 550°C for 4 h.

Figure 1 shows the XRD patterns of Li_2_S, P_2_S_5_, and LiCl powder mixtures obtained after milling (dry and wet) and annealing. For the as-milled powder mixture samples (Figure 1A), characteristic peaks of Li_2_S and LiCl crystalline phases were observed in all the samples, whereas no peaks corresponding to the P_2_S_5_ phase were observed. During the milling process, P_2_S_5_ and Li_2_S formed a Li_2_S-P_2_S_5_ amorphous phase (Hayashi et al., 2001) as suggested by the presence of a halo pattern in the range of 15–20°. Another interesting feature observed was the presence of a peak at 30°, corresponding to Li_6_PS_5_Cl phase, suggesting its mechanochemical synthesis during ball milling [(Boulineau et al., 2012), (Yubuchi et al., 2018)]. This mechanochemical synthesis effect was observed in both dry- and wet-milled powder samples.

In the XRD patterns of the post-annealed powder samples (Figure 1B), the peaks of the Li_2_S and LiCl phases disappeared and the peaks corresponding to the Li_6_PS_5_Cl phase appeared. There was no significant difference between the XRD patterns of the wet-milled and dry-milled Li_6_PS_5_Cl powder samples. Notably, the total synthesis (milling and post-annealing) time in the case of wet-milled and dry-milled samples was 6 and 16 h, respectively. Considering the highly reduced synthesis time to achieve the same composition, it was concluded that wet ball milling is an efficient method to synthesize Li_6_PS_5_Cl powder.

**FIGURE 2:**
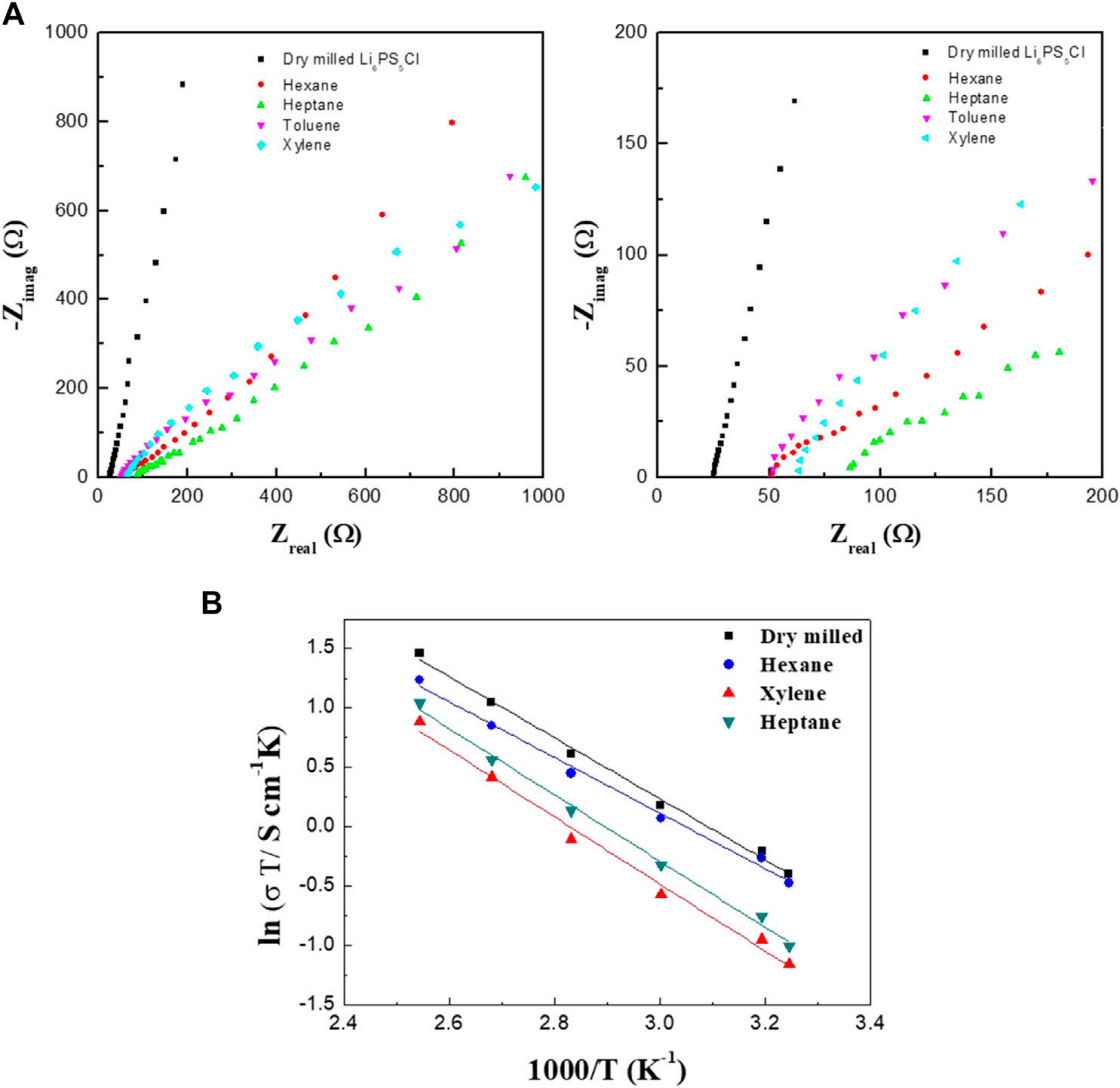
Electrochemical impedance spectra **(A)** and Arrhenius conductivity plot **(B)** of symmetrical cells with wet-milled Li_6_PS_5_Cl electrolytes prepared using hexane, heptane, toluene, and xylene.

**FIGURE 3:**
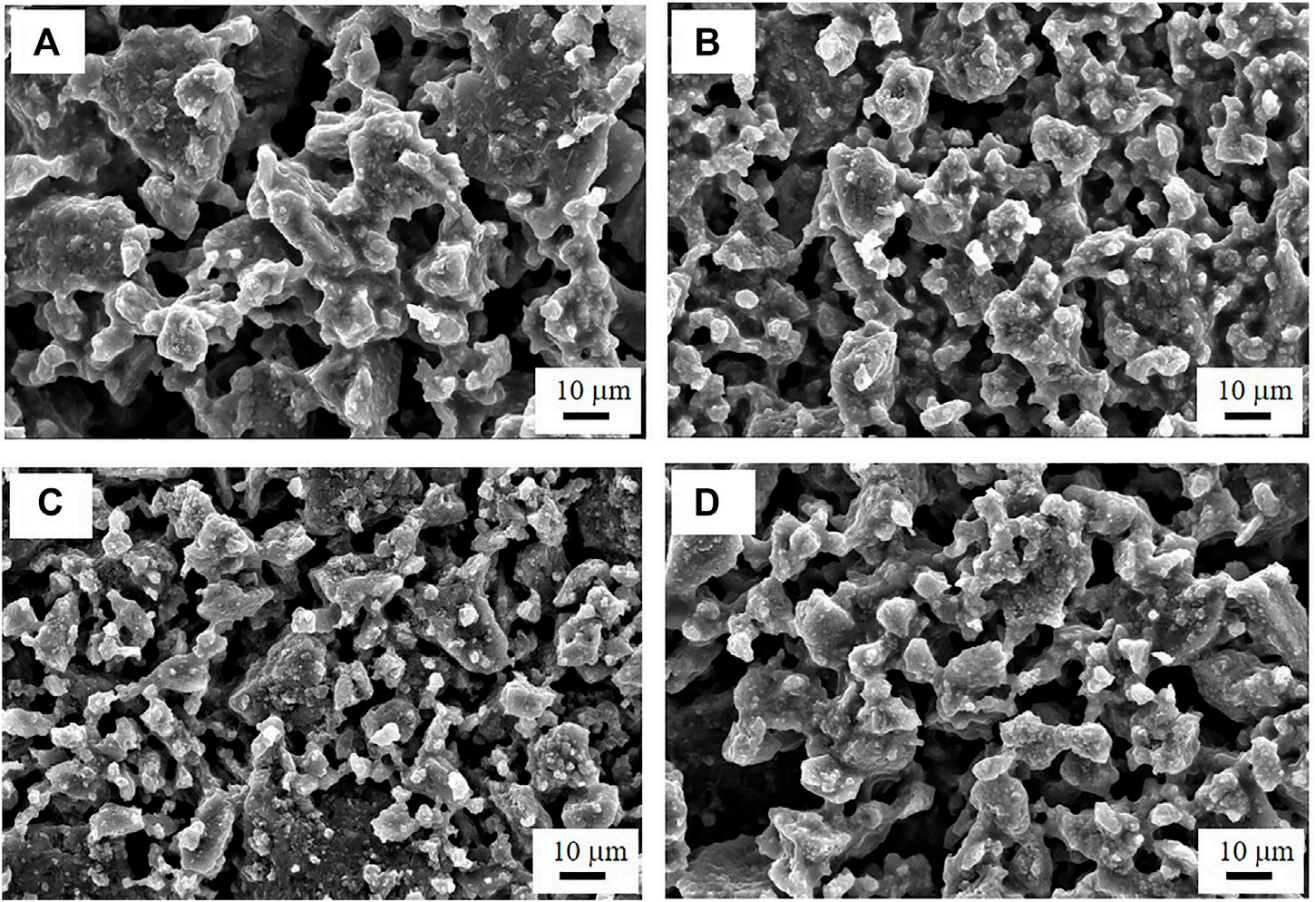
FE-SEM images of the **(A)** dry-milled Li_6_PS_5_Cl powder sample, and wet-milled Li_6_PS_5_Cl powder samples prepared using **(B)** heptane, **(C)** toluene, and **(D)** xylene.

Figures 2A,B show the AC impedance spectra of the SS/Li_6_PS_5_Cl/SS symmetrical cells measured at 25°C. In all the spectra, only the spike (straight line) of the blocking electrodes was observed. The ionic conductivity at room temperature can be derived from the total resistance, which is determined from the intersection of the spike with the real axis (Z_real_) at the lower frequency side. The ionic conductivities of the Li_6_PS_5_Cl electrolyte samples are listed in Table 1. Regardless of the nature of solvent used for the wet milling, the conductivities of the wet-milled Li_6_PS_5_Cl electrolyte pellet samples were estimated to be in the range of 1.0–1.9 mS·cm^−1^. This indicates that the organic solvent does not affect the lithium-ion conduction in the wet-milled Li_6_PS_5_Cl. By contrast, the ionic conductivity of the dry-milled Li_6_PS_5_Cl electrolyte pellet sample was found to be 2.39 mS·cm^−1^, which is slightly higher than that of the wet-milled samples. The observed conductivity reduction in the wet-milled Li_6_PS_5_Cl electrolyte samples could be associated with the particle size (Choi et al., 2019). Yu et al. also have addressed that the grain boundary resistance was reduced in the argyrodite (Li_6_PS_5_Br) electrolyte with large crystallites (Yu et al., 2017). As can be seen in Figure 3, the particles of the wet-milled Li_6_PS_5_Cl powder samples were smaller than those of the dry-milled Li_6_PS_5_Cl powder sample. The apparent small semicircles observed in the low-frequency region of the impedance spectra of the wet-milled Li_6_PS_5_Cl electrolyte samples (hexane and heptane) were due to the grain boundary resistance.

**TABLE 1:** Ionic conductivities Li_6_PS_5_Cl electrolyte pellet samples.

	Wet-milled Li_6_PS_5_Cl				Dry-milled Li_6_PS_5_Cl
	n-hexane	n-heptane	Toluene	Xylene	
Ionic conductivity, mS·cm^−1^	1.94	0.96	1.42	1.49	2.8

**FIGURE 4:**
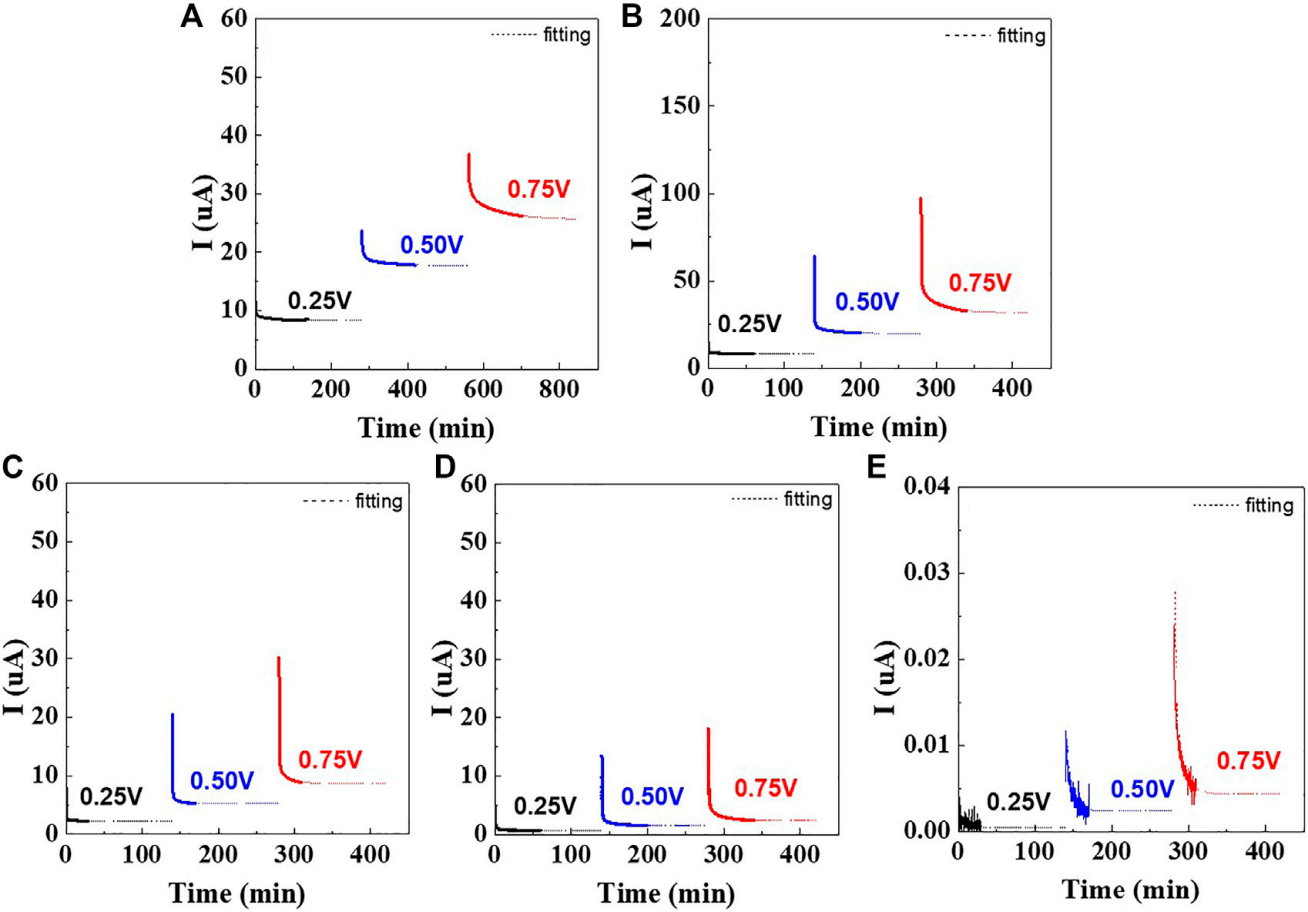
DC polarization curves of symmetrical cells containing wet-milled Li_6_PS_5_Cl electrolytes prepared using **(A)** hexane, **(B)** heptane, **(C)** toluene, **(D)** xylene, and **(E)** dry-milled Li_6_PS_5_Cl electrolyte.

The Arrhenius plots of the ASSCs is shown in Figure 4C. The linear relationship between the reciprocal absolute temperature (1/T) and the logarithm of conductivity (*σ*) was found and the activation energy can be determined using the equation of *σ* = Aexp(−E_a_/k_B_T), where *σ* is the total conductivity, A is the pre-exponential factor, k_B_ is the Boltzmann constant, and T is the absolute temperature. The activation energies are 0.202, 0.240, and 0.245 eV for the hexane, heptane, and xylene-processed Li_6_PS_5_Cl electrolytes, respectively. These values are almost same to the dry-milled Li_6_PS_5_Cl electrolyte (0.221 eV), indicating that there are no unwanted secondary phases in the wet-milled Li_6_PS_5_Cl powder samples, as can be seen in Figure 1.

Figure 4 shows the time dependence of the current for Ni/Li_6_PS_5_Cl/Ni symmetrical cells in which the Li_6_PS_5_Cl powder was prepared using different solvents. When the voltage was applied, the current signal sharply decreased with time and saturated on the order of 10^–5^ to 10^–7^ A, depending on the solvent employed during the wet ball milling process. For all cells, the steady state was achieved within 2 h. The currents of the hexane-, toluene-, and xylene-processed Li_6_PS_5_Cl samples saturated to ∼10^–6^ A, whereas for the heptane-processed Li_6_PS_5_Cl sample, the current saturated to ∼10^–5^ A.

The Ni/Li_6_PS_5_Cl/Ni symmetrical cells exhibit a linear relationship between voltage and steady-state current values (Supplementary Figure S1). In the steady state, the current is carried only by the electrons because of the blocking electrodes. The resulting electronic conductivities are presented in Table 2. The electronic conductivities of the hexane- and heptane-processed Li_6_PS_5_Cl powder samples were calculated to be 2.2 and 2.9 × 10^–6^ S·cm^−1^, respectively, which are one order of magnitude higher than those of toluene and xylene-processed Li_6_PS_5_Cl samples. The electronic conductivities of the wet-milled Li_6_PS_5_Cl samples were three to four orders of magnitude higher than that of the dry-milled Li_6_PS_5_Cl sample. It is noteworthy that impurities and defects in the wet-milled Li_6_PS_5_Cl powder samples can have a negative effect on their electronic conductivity.

**TABLE 2:** Ionic conductivities Li_6_PS_5_Cl electrolyte pellet samples.

	Wet-milled Li_6_PS_5_Cl				Dry-milled Li_6_PS_5_Cl
	n-hexane	n-heptane	toluene	xylene	
Electronic conductivity, S·cm^−1^	1.9 × 10^–6^	3.0 × 10^–6^	6.8 × 10^–7^	2.1 × 10^–7^	4.4 × 10^–10^

To examine the effect of impurities on the electronic conductivity of the Li_6_PS_5_Cl electrolyte samples, elemental analysis (carbon and hydrogen content) of the dry- and wet-milled Li_6_PS_5_Cl powders was performed and the results are presented in Table 3. A higher amount of carbon was detected in the wet-milled Li_6_PS_5_Cl powder samples, which could be attributed to the organic solvents. Due to incomplete evaporation, residual solvent molecules could be deposited on the surface of the wet-milled Li_6_PS_5_Cl powder and reduced to pure carbon during the post-annealing process. These carbon particles located at the grain boundaries of the wet-milled Li_6_PS_5_Cl electrolytes work as electron transport carriers, which explains the increased electronic conductivity. As can be seen in Table 3, the carbon content was lowest in hexane and highest in xylene. However, no distinct relationship between the electronic conductivity and carbon content could be established. The dry-milled Li_6_PS_5_Cl powder sample had the lowest carbon content (0.19%) and exhibited the lowest electronic conductivity of 5.4 × 10^–10^ S·cm^−1^.

**TABLE 3:** Ionic conductivities Li_6_PS_5_Cl electrolyte pellet samples.

Samples	Wet-milled Li_6_PS_5_Cl				Dry-milled Li_6_PS_5_Cl
	n-hexane	n-heptane	Toluene	Xylene	
Carbon,wt%	0,66	1.03	0,73	1.22	0,19
Hydrogen,wt%	0.01	0.06	0.05	0.07	0.06

**FIGURE 5:**
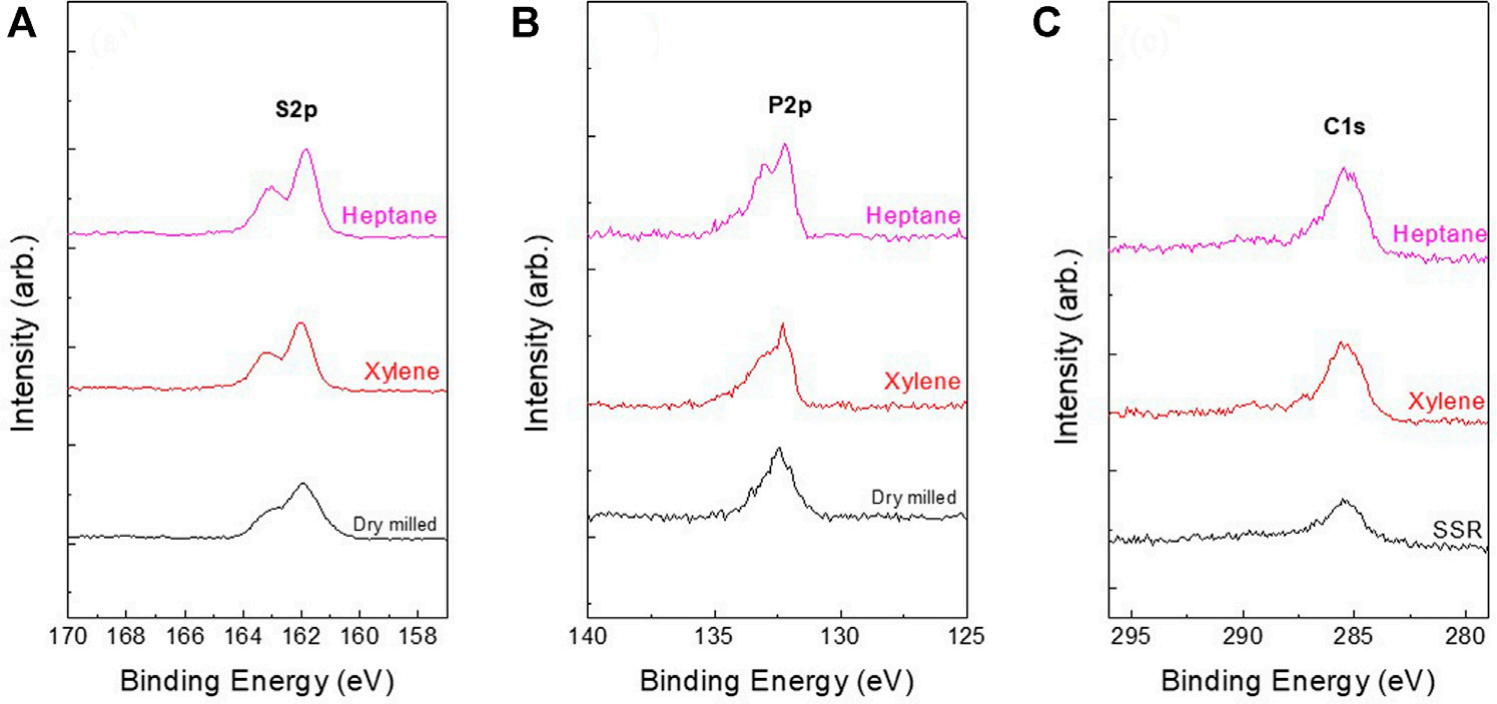
**(A)** S_2p_, **(B)** P_2p_, and **(C)** C_1s_ XPS spectra of dry-milled Li_6_PS_5_Cl and wet-milled Li_6_PS_5_Cl powder samples prepared using hexane and xylene.

Figure 5 presents the S_2p_, P_2p_, C_1s_ and O_1s_ XPS spectra of dry- and wet-milled Li_6_PS_5_Cl powder samples. The S_2p_ spectra of the dry-milled Li_6_PS_5_Cl powder samples exhibited a doublet with a S2p_3/2_ component at a binding energy of 162.2 eV, attributed to the PS_4_^3–^ tetrahedra (P–S–Li bonds) of argyrodite (Walther et al., 2019; Auvergniot et al., 2017). The peaks attributed to the unreacted Li_2_S were not detected in the XPS spectra of S_1s_. Moreover, no sulfur oxides (166–171 eV) or element sulfur (164 eV) were observed (Khallaf et al., 2009). In the P_2p_ spectra, the peak at a binding energy of 132.5 eV was assigned to the PS_4_^3–^ tetrahedra of argyrodite (Yubuchi et al., 2018). In addition, the peaks due to P_2_S_5_ and P_2_S_x_ were not detected in the XPS spectra. This is consistent with the XRD results obtained for the samples (Figure 1). The spectra of dry- and wet-milled Li_6_PS_5_Cl powder samples exhibited no significant differences, indicating that wet milling does not have a detrimental effect on the surface composition of the Li_6_PS_5_Cl electrolyte. In terms of carbon, the samples exhibited two peaks in the C_1s_ region. The lower binding energy peak (∼286 eV) was attributed to the amorphous carbon species (Takahagi and Ishitani, 1988), whereas the higher binding energy peak (∼290 eV) appeared due to the contamination by carbonate species (Bhattacharya et al., 1997). The carbon content values in Table 3 were consistent with the C1s peak area in Figure 5.

**FIGURE 6:**
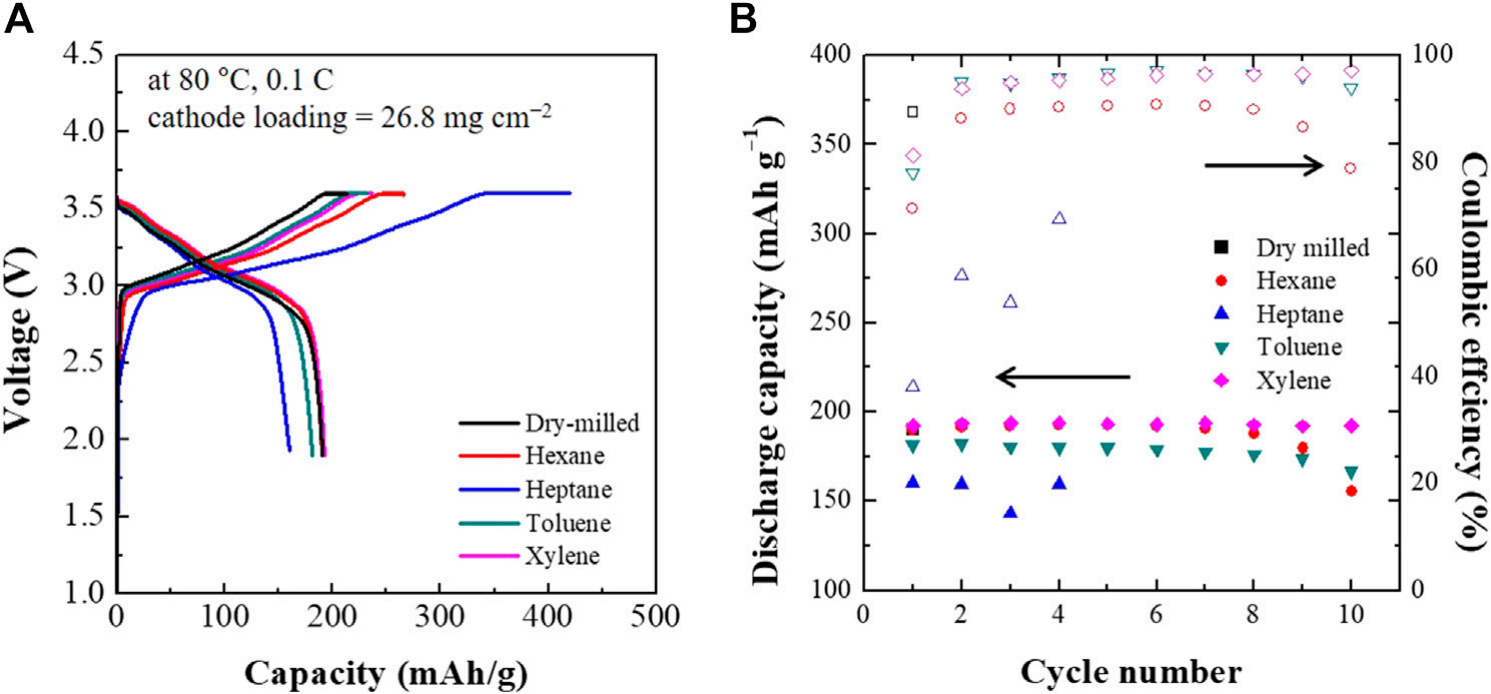
The first charge-discharge voltage profiles **(A)** and cycling performance **(B)** of ASSCs containing dry- and wet-milled Li_6_PS_5_Cl electrolytes.

Figure 6 shows the first charge-discharge voltage profiles and cycling performance of the ASSCs composed of dry- and wet-milled Li_6_PS_5_Cl electrolytes. The first discharge specific capacities for hexane, heptane, toluene, and xylene were 190, 160, 181, and 192 mAh·g^−1^, respectively. ASSCs with the xylene and toluene-processed electrolytes processes the first cycle discharge capacity and coulomibic efficiency comparable to the dry-milled sample. When the xylene-processed Li_6_PS_5_Cl electrolyte was used, the discharge capacity of the ASSC was found to be stable above 190 mAh·g^−1^. Although hexane-processed electrolyte exhibited a higher first discharge specific capacity (190 mAh·g^−1^) than toluene-processed (181 mAh·g^−1^), the latter exhibited better retention of the rate capacity than hexane. The ASSC with the heptane-processed Li_6_PS_5_Cl electrolyte did not maintain its discharge capacity after the fourth cycle.

A different trend was observed for the initial Coulombic efficiencies, as is evident in Figure 6. The Coulombic efficiency of the ASSCs increased in the following order: xylene (81.3%) > toluene (78.1%) > hexane (71.4%) > heptane (38.1%). An interesting feature was that this trend was consistent with the electronic conductivity of the wet-milled Li_6_PS_5_Cl powder samples. In other words, high electronic conductivity can lead to a reduced Coulombic efficiency. The ASSCs with xylene- and toluene-processed Li_6_PS_5_Cl electrolytes maintained a Coulombic efficiency of 95–96% during the first 10 cycles. By contrast, the ASSC with the hexane-processed Li_6_PS_5_Cl electrolytes exhibited Coulombic efficiencies of 90% which gradually decreased with increasing cycle number. In the case of heptane, the ASSC exhibited much lower Coulombic efficiencies than the other ASSCs. The capacity and Coulombic efficiency retention might be closely related to the electronic conductivity of the Li_6_PS_5_Cl electrolyte. The Li_6_PS_5_Cl electrolyte powders prepared using xylene and toluene exhibited relatively low electronic conductivity on the order of 10^–7^ S·cm^−1^, resulting in high discharge capacity and Coulombic efficiency retention after 10 cycles, whereas those prepared using hexane and heptane, exhibited higher electronic conductivities, lower discharge capacity and decaying Coulombic efficiency with cycling.

## Conclusion

A single-phase Li_6_PS_5_Cl powder was synthesized from Li_2_S, P_2_S_5_, and LiCl powder mixture via wet ball milling with various organic solvents and post-annealing. Compared to dry milling, the processing time was significantly reduced. The ionic conductivities of the wet-milled and post-annealed Li_6_PS_5_Cl powder samples were in the range of 1.0–1.9 S·cm^−1^, which was slightly lower than that of the dry-milled Li_6_PS_5_Cl powder sample (2.8 S·cm^−1^). FE-SEM analyses revealed that the particle size was reduced in the wet-milled Li_6_PS_5_Cl powder samples, and the increased grain boundary was responsible for the reduced ionic conductivity. The wet-milled Li_6_PS_5_Cl powder samples showed three or four orders of magnitude higher electronic conductivity than that of the dry-milled Li_6_PS_5_Cl powder sample. In terms of solvent for wet milling, xylene and toluene were found to be better than hexane and heptane, leading to one order of magnitude lower electronic conductivity. It appears that sulfur deficiency or the state of the residual carbon could be responsible for the observed variation in the electron conductivity. The ASSC with the xylene-processed Li_6_PS_5_Cl electrolyte exhibited the highest discharge capacity of 192.4 mAh·g^−1^ and a Coulombic efficiency of 81.3% for the first discharge cycle. The lower discharge capacity and Coulombic efficiency retention observed in the ASSCs with hexane- and heptane-processed Li_6_PS_5_Cl electrolytes was attributed to the increased electronic conductivity of these electrolytes. In the future, the strategies to reduce the electronic conductivity of the wet-milled Li_6_PS_5_Cl should be presented in order to use it as an all-solid-state lithium battery electrolyte.

## Data Availability

The raw data supporting the conclusions of this article will be made available by the authors, without undue reservation.
