# Supplementary figures and images for: Ionic and Electronic Conductivities of Lithium Argyrodite Li6PS5Cl Electrolytes Prepared via Wet Milling and Post-Annealing
Source: Front Chem. 2021 Dec 16;9:778057. doi: 10.3389/fchem.2021.778057 (PMC8717468; doi:10.3389/fchem.2021.778057)

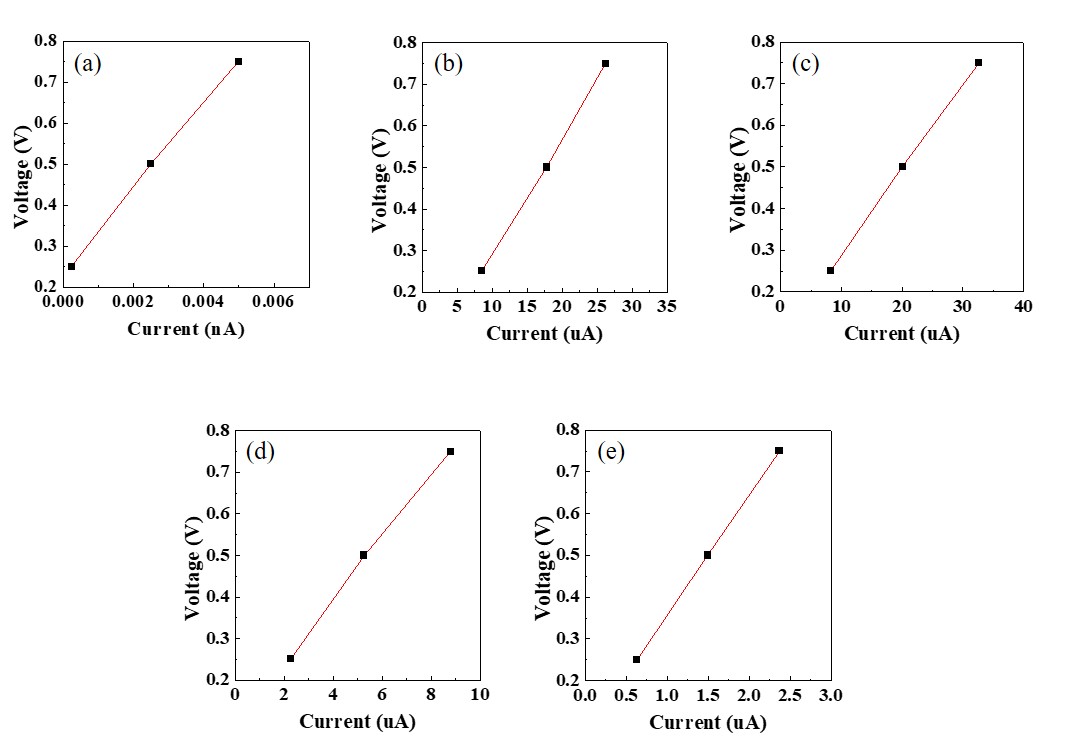

Supplement: Supplementary file 1 [file Image1.jpg]
